# Assessing the perspectives of users and beneficiaries of a community health worker mHealth tracking system for mothers and children in Rwanda

**DOI:** 10.1371/journal.pone.0198725

**Published:** 2018-06-07

**Authors:** Angele Musabyimana, Hinda Ruton, Erick Gaju, Atakilt Berhe, Karen A. Grépin, Joseph Ngenzi, Emmanuel Nzabonimana, Celestin Hategeka, Michael R. Law

**Affiliations:** 1 School of Public Health, College of Medicine and Health Sciences, University of Rwanda, Kigali, Rwanda; 2 The Centre for Health Services and Policy Research, School of Population and Public Health, The University of British Columbia, Vancouver, Canada; 3 Ministry of Health, Kigali, Rwanda; 4 UNICEF Rwanda, Kigali, Rwanda; 5 Department of Health Sciences, Wilfrid Laurier University, Waterloo, Ontario, Canada; 6 School of Dentistry, College of Medicine and Health Sciences, University of Rwanda, Kigali, Rwanda; 7 Collaboration for Outcomes Research and Evaluation, Faculty of Pharmaceutical Sciences, UBC, Vancouver, BC, Canada; 8 Department of Global Health and Social Medicine, Harvard Medical School, Boston, MA, United States of America; Columbia University, UNITED STATES

## Abstract

**Introduction:**

Mobile Health (mHealth) programs have increasingly been used to tackle maternal and child health problems in low and middle income countries. However, few studies have evaluated how these programs have been perceived by intended users and beneficiaries. Therefore, we explored perceptions of healthcare officials and beneficiaries regarding RapidSMS Rwanda, an mHealth system used by Community Health Workers (CHWs) that was scaled up nationwide in 2013.

**Methods:**

We conducted key informant interviews and focus group discussions with key stakeholders, providers, and beneficiaries of maternal and child health services at both the national and community levels. Semi-structured interviews were used to assess perceptions about the impact of and challenges facing the RapidSMS system. Interviews and focus group discussions were recorded (with the exception of one), transcribed verbatim, and analyzed.

**Results:**

We conducted a total of 28 in-depth interviews and 10 focus group discussions (93 total participants). A majority of respondents believed that RapidSMS contributed to reducing maternal and child mortality rates. RapidSMS was generally accepted by both CHWs and parents. Participants identified insufficient training, a lack of equipment, and low CHW motivation as the main challenges facing RapidSMS.

**Conclusion:**

Our findings suggest that an mHealth program can be well accepted by both policymakers, health providers, and the community. We also found significant technical challenges that have likely reduced its impact. Addressing these challenges will serve to strengthen future mHealth programs.

## Introduction

Improving maternal and child health outcomes in low and middle-income countries remains a major challenge. Globally, the United Nations estimated that the maternal mortality ratio (MMR) was 216 per 100,000 births and the under-five mortality rate 43 per 1,000 live births in 2015 and that the majority of these deaths occurred in Sub-Saharan Africa [1–3]. An estimated 75% of maternal deaths and more than 80% of newborn deaths worldwide are preventable [4,5].

Rwanda has seen considerable improvement in maternal and child health outcomes over the last decade; theMMR decreased from 750 per 100,000 births in 2005 to 210 in 2014–15 and under-five mortality from 152 deaths per 1,000 live births to 50 over the same time period[6,7]. Utilization of maternal and child health services is high in Rwanda, as 91% of pregnant women delivered at a health facility compared to 49.6% for the Africa region [6,8]. Despite these reductions in mortality and high rates of health service utilization, maternal and newborn mortality remain high, and most of the remaining deaths are preventable. For instance, a recent study showed that 74% of newborn deaths were due to delays in seeking care and health care workers not providing appropriate care [9]. As a result, initiatives to try and enhance the link between the population and formal health care services remain a priority in Rwanda.

As in many other countries, Rwanda has implemented technical innovations to try and improve maternal and child health outcomes. Specifically, there has been an increasing number of health applications using mobile phones, also known as mHealth initiatives. Due to the high penetration of mobile phones in Africa, mHealth has considerable potential to reach many individuals, even in settings with limited infrastructure and human resources [10,11]. In fact, the combination of a near-universal access to mobile phone, a near global mobile phone network coverage and a considerable reduction in devices and communication cost has made mHealth a strategy of choice in improving public health by collecting data and communicating with health providers and the general population [12].

Rwanda’s key mHealth program in this area, known locally as RapidSMS, aimed to improve access to antenatal, postnatal care, institutional delivery and emergency obstetric care. The RapidSMS system has been previously described elsewhere [13]. In brief, RapidSMS was designed to send automated reminders for clinical appointments to community health workers (CHWs), collect important data on child development, and facilitate two-way communication with the health facility for emergency obstetric care. Following piloting in one district, RapidSMS was scaled-up nationwide in 2013 to all public health facilities in Rwanda.

Despite the rapid growth in the number of mHealth programs globally, few programs have been scaled-up nationally and even fewer have been qualitatively assessed the perceptions of intended users and beneficiaries [11,14–16]. We evaluated RapidSMS program using a mixed methods approach. The quantitative component, reported elsewhere, assessed the impact of RapidSMS program on the use of key maternal and child health services in Rwanda. For the qualitative component of the evaluation, which is reported here, our aim was to explore perceptions of healthcare officials, providers, and beneficiaries on the impact of the RapidSMS program, identify challenges, and make suggestions for modifications to the program moving forward.

## Methods

### Study context

The RapidSMS platform was implemented by the Rwanda Ministry of Health (MoH) to facilitate communication between CHWs and the broader health system, including the ambulance system, health facilities, and MoH officials. CHWs provide care at the community level, and offer preventive and basic curative services [17]. They are intended to be the first point of contact with the health system for the population and to create a liaison between community health services and health facilities. Each village elects three volunteer CHWs, of which one is specifically tasked with maternal and child health care. CHWs are required to have at least 6 years of formal education. As part of the program, CHWs were equipped with mobile phones that enabled them to collect and receive real-time data on key maternal, neonatal and child health indicators for women and children in their catchment area by SMS.

The RapidSMS system send automatic reminders to CHWs for clinical appointments, delivery, and post-natal care visits, with the intent of increasing attendance at antenatal care, postnatal care visits and institutional delivery. Additional intended outcomes include the provision of a quick link to emergency obstetric care through so-called Red Alerts, and the creation of a database of clinical records on maternal care delivery [18]. Importantly, 10 of Rwanda’s 30 administrative districts received additional support to improve maternal and child health consisting of additional training, the provision of equipment, and supervision. The 10 administrative districts that received additional support will be referred below as supported districts. The nature of this support has previously described in more detail [19].

### Study population

As part of a broader impact evaluation of the program [19], we also interviewed officials within national institutions (e.g. MoH) and district officials, healthcare providers, CHWs and parents in four selected districts. We selected two districts in supported districts and two in non-supported ones. The selection criteria we considered while sampling and selecting facilities to be visited included their performance in maternal and child health, location (urban versus rural), the level RapidSMS program use, and the geographical accessibility of clients to the health facilities.

Targeted participants for interviews were identified in a by the research team and validated by a steering committee composed with Ministry of Health officials and stakeholders working in the domain of maternal and child health in Rwanda. contacted directly by members of the research team and invited to participate. Those who agreed were given an appointment for an interview, which was semi-structured in nature. Participants in the focus groups were recruited through the CHWs supervisor at each health center. This included both CHWs and beneficiaries (mothers and fathers) who had been provided with their services during a pregnancy or partner’s pregnancy. After identification, verbal invitations were sent to them through CHWs specifying the venue and time of the focus group.

### Data collection procedures

Our research team developed semi-structured interview guides in both English and Kinyarwanda, including one for each category of focus group and interview S1 File. If consent to record was given, research team members (AM and HR) made a recording of each interview and focus group. In addition, two team members (JN and EN) made notes regarding the responses during the interview and two field workers during focus groups. Interviews were conducted in either Kinyarwanda or English, depending on the preference of the interviewee(s). Interviews were conducted at the venue chosen by the interviewee, and focus groups were conducted in health center’s meeting rooms.

### Data management and analysis

After each interview or focus group, the note taker transcribed verbatim all interview and focus group discussion content. To ensure the quality of the resulting data, two interview transcripts and two focus group discussion transcripts were randomly selected and quality-checked by one of the study investigators (AM). The resulting comments were shared with note takers and supervision was conducted to ensure all field notes and transcripts were of high quality.

Our analysis of the data proceeded in four steps. First, we reviewed our field notes to be familiar with their contents; this was paired with the data collection activities in which major themes were identified. Second, an initial set of codes were created from interview and focus group transcripts, which were further enriched with detail throughout the coding process. Third, AM and HR proceeded to code all transcripts and field notes using ATLAS.TI. Finally, after codification was completed, we produced output on specific themes/codes S2 File. We performed a content analysis. The analysis of transcripts was conducted in the language in which the interviews and focus groups were conducted. When the original interview or focus group discussion was conducted in Kinyarwanda, specific quotes were translated to English by the investigators for inclusion in our results. Our research protocol was approved by the University of Rwanda College of Medicine and Health Sciences Institutional Ethics Review Board.

## Results

### Study participants

Of the 32 individuals targeted for an in-depth interview, 28 agreed to participate and were available for an interview. Of these, 10 were from national level organizations, including Ministry of Health officials, academics, representatives of professional bodies, and developing partners. The remaining 18 were from the four selected districts, including two District Health Unit (DHU) members, four CHW supervisors at District Hospital level, four healthcare providers and four CHW supervisors from each health center, and four CHW coordinators at the administrative cell level.

We conducted all 10 planned focus groups, which included 93 participants (the minimum number of participants in a focus group was 7 and the maximum 12). A total of 56 beneficiaries (39 mothers and 17 fathers) and 36 CHWs (33 females and 3 males) were interviewed. As planned, we conducted four focus groups with CHWs, four focus groups with mothers and two with fathers.

### Acceptance of RapidSMS by the community

Generally, participants at the district level along with beneficiaries felt that RapidSMS was well accepted by most CHWs and community members. It was also mentioned that CHWs like the system because it increased the quality of their collaboration with community members. Members of the community also indicated they valued the speed with which messages could be sent in comparison with the time a formal appointment at the health center would consume. Though this was the general perception, we also heard that there was a small proportion of community members who have a negative view of the system.

“They accept it! Now you will find women who take the initiative to look for a CHW to share the information that she is pregnant. Some also ask the CHW if the information about the fact that she is pregnant was sent through RapidSMS! After delivery, some also remind the CHW to send a message about the event. But this is not yet 100% of women.”

- ***CHW supervisor at HC level in a District not supported by UNICEF***

*“CHWs are very pleased to use the system*! *They are happy to see how they are assisting women and women are asking them to send both common and alert messages for their emergency cases*.*”—*CHW supervisor at HC level in a UNICEF supported District

*“We are motivated to use the system*. *However*, *some community members have a poor mindset regarding RapidSMS thinking that we are selling their information and getting paid for the information we send*. *As a result*, *sometimes they hide the needed information*.*”*—CHW from a District not supported by UNICEF

*“I can see we dropped maternal mortality we reached MDG*, *about maternal reducing maternal mortality we reached MDG… and the impact is very positive for me and may be statistically you can’t report what is related to RapidSMS only because there were so many interventions in the area*. *But generally it is contributing so much toward the reduction of maternal and newborn and child mortality*.*”*—Central level participant

*“RapidSMS has helped a lot to prevent maternal*, *child and neonatal death*. *Through the collaboration between the health facility and CHWs and the information shared with RapidSMS*, *once the mother is reminded and attends the needed service on time*, *providers are motivated to do their best to keep the newborn and the mother alive”*.*—*CHW from a district not supported by UNICEF

*“What I can say*, *it was generally improved*. *Before 2012*, *maternal and child mortality was high*, *but nowadays*, *it is reduced”*.—Male parent from a UNICEF supported district

### Challenges

Many challenges faced by RapidSMS were mentioned across the different categories of participants. The main cited four areas with challenges impacting the functionality of RapidSMS are highlighted below. However, it is worth noting that there is a difference of perceptions between participants from supported and non-supported districts.

#### 1. Training of community health workers

CHWs appreciated the initial training they received when the system was launched. It was well prepared and contained enough information to allow them to start the RapidSMS reporting process. CHWs from supported districts appreciated refresher trainings received to keep their skills up to date. However, CHWs in non-supported districts expressed frustration that refresher trainings were not available to them. This left them responsible for training and assisting new CHWs when they start.

*“Last year*, *they (CHW) were trained on a quarterly basis on how to use RapidSMS*. *We were supported by UNICEF*. *They were trained last quarter for a few days*.*”*—CHW supervisor at DH level in a UNICEF supported District

*“We were trained only once*. *The challenge we have is that we work with colleagues who were never trained”*.—CHW from a District not supported by UNICEF

The need for training was particularly noted innutrition. Notably, both CHWs and their supervisors reported confusion about the malnutrition indicators in RapidSMS.

*“CHWs take measurements of the child …Okay*! *They have the tools*, *they take the measurements*, *then they classify children as “red” or “yellow”*. *But the indicators used confuse them*. *They mix them up*: *some use mid-upper arm circumference (MUAC)*, *others weight for age*, *weight for height*, *… there is a need to have a look on how to train them better or maybe there is a need to recruit more qualified personnel*. *There is a need for improvement*.*”***—**District official participant from a UNICEF supported District

#### 2. Equipment availability to community health centers

In supported districts, equipment was provided to CHWs and community health centers to ensure that proper measurements could be taken and entered into the RapidSMS system. In non-supported districts, on the other hand, we heard reports from some CHWs that they lacked the necessary equipment to report nutrition-related outcomes requested in RapidSMS. For example, they reported not having tools to measure middle upper arm circumference, temperature, height, and weight.

*“Equipment are not yet available… We need the right equipment to take measures from pregnant women and children*. *For example*, *we need instruments to measure the height and weight of pregnant women because those measures were not taken at the health facility…*. *We also need thermometers to measure temperature*.*”—*CHW from a non-supported District

#### 3. Motivation of community health workers

Providing appropriate motivation to CHWs was identified as an important challenge for RapidSMS in supported and non-supported districts. CHWs argued that financial compensation would both increase the reporting rate for patients and to help retain CHWs. Participants indicated that the lack of motivation might impact turnover rates.

*“CHWs need more motivation [for using RapidSMS]*. *Their motivation is dropping with time*. *I think this lack of motivation may increase their turnover rate*.*”*—CHWs supervisor at DH level in a UNICEF supported District

*“We know that we are volunteers and RapidSMS necessitates a lot of community-based work*. *It seems like there should be a financial motivation for all this work*, *but we do not receive it*. *… If we were paid for our time*, *we would be more motivated to work as CHWs because there would be compensation to help support our families*.*”*—CHW from a District not supported by UNICEF

#### 4. Improve technical aspects of phone service

We heard several comments from CHWs and their supervisors regarding difficulties with using the provided phones and mobile services. For example, several expressed difficulties with the phones which were provided as part of the program.

*“In addition*, *the phones given to CHWs are old*. *Now they are buying phones using their own money*. *Providing them with new phones would provide them with a type of motivation*.*”*—CHW supervisor at HC level in a UNICEF supported District

### The future of RapidSMS

In both our interviews and focus groups, participants gave many suggestions for improving the RapidSMS system in the future.

#### 1. Provide direct messages to beneficiaries

Several respondents suggested that the system be modified to directly message with beneficiaries. For example, if woman were linked directly to the system, this would allow them to directly receive reminder messages. Another possibility that was mentioned was that women might also use the system to request an appointment at her health facility if needed. In an interview with the RapidSMS administrator, we were informed that, currently, the system has the capabilities to send the message directly to beneficiaries.

*“I wonder whether pregnant women should provide their phone number*, *since they are the beneficiaries*. *Then both she and the CHW would receive the RapidSMS messages*. *… This system would help remind parents; we are very busy and we sometimes forget all our responsibilities to our children”*. -Father from an urban area

*“Through RapidSMS*, *I think it should be possible for someone in a village to exchange information with their community health worker or other health professionals on their child’s health in direct way*. *I suspect this step would take some time to be implemented*, *but we should start planning it*. *Secondly*, *patients might be able to use it to request an appointment at the health center*. *This would help someone living a long distance from the health center*, *as they could use RapidSMS to ensure they have an appointment*. *Health care providers could also then use it to see the order of consultations for the day*. *This would reduce the time patients spend queuing for an appointment*.*”—*Central level participant

#### 2. Improve access to RapidSMS data

Several health care providers and district-level staff mentioned that it would be better if they had direct access to RapidSMS data. They said that this would help them to plan and make timely decisions about their daily duties.

*“We don’t have access to the information in RapidSMS*. *The data manager and the CHW supervisor are the only ones who have access to this information*. *For example*, *we would like to be able to check whether all the women who delivered at our health center were registered in RapidSMS*, *because we have their name in our registers*.*”-* Provider from a District not supported by UNICEF

*“I wish that staff working at the district would have access to RapidSMS data*. *For example*, *the director of health unit*, *M&E officer*, *and the CHW supervisor at the district hospital*. *They are all very busy*, *so may not have enough time to consult with a data manager every time they want to use data for decision making*.*”-* DHU member from a District not supported by UNICEF

#### 3. Build capacity for analyzing RapidSMS data

Also with respect to data, respondents related that there is a limited capacity for properly using and analyzing the data from RapidSMS. This was blamed on a lack of capacity in analysis skills among the people who are supposed to analyze the RapidSMS data.

*“What needs to be improved is the use of the data we are collecting*. *Health facility staff and everyone who has access to RapidSMS should be trained on data analysis so that they can benefit from the system and know what type of information is provided by the system*.*”*—CHW supervisor at DH from a District not supported by UNICEF

### Impact of RapidSMS

Most healthcare officials (from CHW to Ministry of Health officials) stated that RapidSMS contributed to reducing maternal and child mortality rates in Rwanda. Further, there was consistent sentiment that the system contributed more generally to improvements in the quality of services provided both in the community and in health facilities. For example, district-level authorities as well as health center and community level providers mentioned that RapidSMS has helped them to reach their targets for care provision. The one caveat noted by several participants was that RapidSMS was not the only program during this time that could have contributed positively to maternal and child health improvements.

*“I can see we dropped maternal mortality we reached the millennium development goal(MDG)*, *about maternal reducing maternal mortality we reached MDG… and the impact is very positive for me and may be statistically you can’t report what is related to RapidSMS only because there were so many interventions in the area*. *But generally*, *it is contributing so much toward the reduction of maternal and newborn and child mortality*.*”***—**Central level participant

*“RapidSMS has helped a lot to prevent maternal*, *child and neonatal death*. *Through the collaboration between the health facility and CHWs and the information shared with RapidSMS*, *once the mother is reminded and attends the needed service on time*, *providers are motivated to do their best to keep the newborn and the mother alive”*.—CHW from a non-supported district

In several instances, study participants expressed that RapidSMS data was helpful for health system planning purposes. For example, in a non-supported district, hospital staff have been using the data collected through RapidSMS to plan surgeries. RapidSMS data were used to identify patients with cleft lip and/or cleft palette for program such as Operation Smile comes to Rwanda. Similarly, health center staff in a supported district use RapidSMS data for planning and decision making in their weekly meetings.

*“…through RapidSMS I know when there is a newborn with this congenital abnormality and what health center they use*. *When this happens*, *we can initiate contact with the hospital and physicians who perform the repair procedures for such children*. *… I get this information and can act without even leaving my office*.*”-* CHW Supervisor from a district not supported by UNICEF

*“In our Monday staff meetings*, *we share information from the RapidSMS system with departments that can use it*. *For example*, *we give department-related information to the head of maternity care unit at the health center*. *The same is done for immunization services*, *and so on…”—*Health center staff in a UNICEF-supported district

Despite the opinion of most participants that RapidSMS was having an impact, there were some participants who felt the impact of RapidSMS on antenatal care (ANC) and assisted delivery was likely to be minor. These respondents felt this resulted from mothers who did not follow the reminders, and those who were not attending antenatal care entirely.

*“Mothers who deliver at home are those who are not reported*. *When some mothers are informed that it is time to deliver they dismiss the CHW and would later deliver at home*. *We have discussed at least two specific women who were informed that they have to go to the health facility*, *but dismissed the CHW stating that it is not yet time*, *we will be going there later”-* DHU member from a District not supported by UNICEF

*“There are mothers who are not attending antenatal care until they come for delivery*. *You wonder in that kind of case how that mother has not been located or identified until the time of delivery*.*”*—Academic participant

## Discussion

As the use of mobile phones has become widespread, mHealth programs are been increasingly used to target health problems in many low- and middle-income countries. We found that the RapidSMS program in Rwanda was widely accepted by policymakers, healthcare providers, and program beneficiaries. We found, also, that participants believed that RapidSMS played a role in improving maternal and child health outcomes. At the same time, participants identified several challenges that potentially hindered the program from achieving its objectives. Motivation of CHWs was the most cited by participants from supported and non-supported districts.

Our quantitative analysis of RapidSMS appears to give credence to these concerns. In our interviews, we found a high-level agreement that RapidSMS had an impact in reducing maternal and child mortality. The causal impact of RapidSMS was unclear, however, as several participants noted several other programs targeting maternal and child health issues were implemented during the same period. In our quantitative analysis of the impact of RapidSMS using interrupted time series analysis, we found that, overall, the program had limited impact, but did show some impact on selected indicators in districts that received additional support [19]. For example, when combined with additional support and equipment, however, it was associated with increased delivery in health care facilities, and increases in post-natal care visits.

Our results reinforce prior findings that mHealth programs are acceptable to beneficiaries across a range of different programs [20]. We found that RapidSMS was generally well accepted by the community, in particular by CHWs. Beneficiaries reported that they felt that the Ministry of Health knew them personally and CHWs that its cared about their work. A growing body of knowledge has indicated that overwhelmingly, beneficiaries of mHealth program are willing to interact with the health system using mobile phones. More than 90% of interviewed mothers indicated willingness to receive SMS reminders, in studies conducted in the North America, Asia and Africa [21–24]. Overall, this is promising for the future development of mHealth programs.

These programs take resources to run, however, and a shortage of resources was a distinct challenge noted by several interview participants. Most notably, a decline in the motivation of CHWs was identified by participants as one of the main challenges facing RapidSMS. As CHWs are volunteers in Rwanda, the necessity of reporting data into the RapidSMS system led to an increase in their task load, and led CHWs to note that financial compensation could help further the program. Whether this led or will lead to a decrease in tenure remains unknown. In conjunction with this increased workload, CHWs noted a lack of ongoing training, and lack of proper equipment appeared to increase the challenges for CHWs. These are similar factors to those identified in motivating CHWs in a prior study conducted across 3 sub-Saharan Africa countries [25].

### Limitations

There are some limitations to our analysis that deserve mention. In order to recruit CHWs and beneficiaries for our focus groups, we relied on the CHW manager within each health facility. While this facilitated recruitment of a sufficient number of participants, it remains unknown if they applied some particular selection criteria to individuals thus reducing the thematic saturation of our sample. For example, they may have selected the most effective or engaged CHWs to participate. However, we heard very similar themes across different Districts, making us more confident this was not the case. Also, it is unclear whether central level participants may have expressed opinions that were subject to social desirability bias given their role in the development of the program. Finally, while we were quite successful in securing interviews with the desired individuals from the Central Level and Districts, we were unable to secure an interview with one of the key developing partners, meaning that perspective was not incorporated into our results.

## Conclusions

As with other research on mHealth programs, we found that RapidSMS system was widely accepted in Rwanda, and beneficiaries believed it had a positive impact on maternal and child health. However, participants identified systemic challenges which might impede the success of both this and other mHealth systems, including the lack of support, training, equipment, and sufficient motivation on the part of CHWs. This suggests that mHealth interventions need to be implemented with the necessary health system capacity for them to maximize their impact.

## Supporting information

S1 File Code book RapidSMS. (DOCX)

S2 File Interview guides. (PDF)
