# Supplementary material for: Assessing the perspectives of users and beneficiaries of a community health worker mHealth tracking system for mothers and children in Rwanda
Source: PLoS One. 2018 Jun 7;13(6):e0198725. doi: 10.1371/journal.pone.0198725 (PMC5991741; doi:10.1371/journal.pone.0198725)
Supplement: S1 File — (DOCX) [file pone.0198725.s001.docx]

**RapidSMS evaluation codebook**

| **Theme** | **Code (Sorted thematically)** | **Code Definition** |
| --- | --- | --- |
| Considerations about the role ofacademia and community as stakeholders **(Emerging theme)** | Academia role in RapidSMS | This code refers to the information shared by participants about what could be the role of academia people in designing, sharing information about and evaluating RapidSMS evaluation as well as what is the real role they play. |
|  | Beneficiaries role in RapidSMS | This code refers to the information shared by participants about what could be the role of community for the smooth implementation of RapidSMS and what they really do. |
| Perceived impact of RapidSMS | Changes prompted by RapidSMS | This code refers to the information shared by participants about what what changes are thought to be the results from RapidSMS use in service delivery or other area which are not maternal and child health. |
|  | No impact area | This code refers to the information shared by participants about areas where RapidSMS was intended to have an impact but it had not. |
|  | RapidSMS impact | This code refers to the information shared by participants about the perceived impact of RapidSMS on maternal and child health. |
|  | RapidSMS effectiveness | This code refers to the information shared by participants regarding general achievements through RapidSMS compared to what the system was intended to achieve. |
| Acceptability of RapidSMS among CHWs and general community **(Emerging theme)** | Perceptions about RapidSMS | This code refers to the information shared by participants regarding how the general community and CHWs perceive RapidSMS, to which extent they find it important and are happy with it. |
| Challenges | Hard to report groups | This code refers to the information shared by participants regarding which specific group whose information is hard to get in order to be reported in RapidSMS. |
|  | RapidSMS training | This code refers to the information shared by participants regarding all challenges in RapidSMS use that are related with CHW training. |
|  | RapidSMS use challenges | This code refers to the information shared by participants regarding all other challenges faced by RapidSMS users as well as coordinators. |
| Ways to overcome challenges | Helping hard to report people | This code refers to the information shared by participants about how to make comfortable the “hard to report groups” members and tracking them |
|  | Ways to overcome challenges | This code refers to the information shared by participants regarding all other ways to be considered for addressing challenges that are faced by RapidSMS users as well as coordinators. |
| Training regarding Rapid SMS | CHWs' perceptions about training on RapidSMS | This code refers to the information shared by participants regarding how CHWs perceive received initial training on RapidSMS, to which extent they find pertinent and are happy with it. |
|  | RapidSMS refresher training | This code refers to the information shared by participants regarding how CHWs perceive received refresher training on RapidSMS, to which extent they find pertinent and are happy with it. |
| RapidSMS coordination | RapidSMS scale-up | This code refers to the information shared by participants regarding on which basis the RapidSMS scaling up was decicded. |
|  | Reporting rate | This code refers to the information shared by participants regarding what is the report rate compared to what was expected. |
|  | RapidSMS efficiency | This code refers to the information shared by participants regarding the financial and human resource management in RapidSMS |
|  | RapidSMS data use | This code refers to the information shared by participants regarding how RapidSMS data are used in planning and decision making at different levels of health system. |
|  | RapidSMS and equity | This code refers to the information shared by participants regarding how the starting point of RapidSMS focused or not on most deprived areas, areas with high prevalence of critical newborn and under-5 mortality, low income families. |
|  | RapidSMS coordination and management | This code refers to the information shared by participants regarding general consideration about RapidSMS coordination and management |
|  | RapidSMS relevance | This code refers to the information shared by participants regarding describing to what extent has RapidSMS contributed to national developmental targets, health included. |
|  | RapidSMS integration | This code refers to the information shared by participants regarding the general integration of RapidSMS in health system (financially, human resources, existing intervention in community…). |
| Future of RapidSMS | RapidSMS sustainability and ownership | This code refers to the information shared by participants regarding what is being done and/or could be done for RapidSMS sustainability and ownership by the Government. |
|  | Needed improvements | This code refers to the information shared by participants regarding what areas to be improved for the better future of rapidSMS. |
| Added value of RapidSMS in CHW work **(Emerging theme)** | Role of RapidSMS in CHW work | This code refers to the information shared by participants regarding what is the perceived improvement of the work environment of CHWs brought by RapidSMS. |
| How the community is informed about RapidSMS **(Emerging theme)** | Source of information about RapidSMS among beneficiaries | This code refers to the information shared by participants regarding how community was informed about RapidSMS. |
