# Supplementary material for: Assessing the perspectives of users and beneficiaries of a community health worker mHealth tracking system for mothers and children in Rwanda
Source: PLoS One. 2018 Jun 7;13(6):e0198725. doi: 10.1371/journal.pone.0198725 (PMC5991741; doi:10.1371/journal.pone.0198725)
Supplement: S2 File — (PDF) [file pone.0198725.s002.pdf]

Interview Guide

Central Level and Developing Partners

Research Project: Evaluating the impact of RapidSMS

Principal Investigator: Michael Law, PhD

University of Rwanda-College of Medicine and Health Sciences/ School of Public Health, Kigali

Respondent Information

1. Name:
2. Organization:
3. Title (describe his/her role): ………
4. Gender:
5. Level of education:
6. Present position:
7. Number of years and Experience at the current position:
8. Previous organization where s/he worked, position, and number of years and roles:

   ………

General topics to be discussed during the interview

I. Introduction questions

1. What are your responsibilities related tomaternal and child health?

2. How were you involved in the development of the RapidSMS system?

3. How are you currently involved in the coordination or management of the RapidSMS System?

II. RapidSMS design and implementation

4. What do you think the major objectives of RapidSMS were when it started?

5. How were decisions made about where and when to implement RapidSMS?

Probe 1: How did equity considerations play a role in these decisions?
Probe 2: What factors were part of the decision to scale-up to a national scale?

6. What, if any, challenges were faced in implementing the RapidSMS system? How were these challenges overcome?

7. What challenges is the RapidSMS system facing now?

8. What are your perceptions about the amount of financial and human resources which were allocated to RapidSMS?

Probe: Inquire about specific human and financial resources for coordination, awareness, training, and supervision.

9. What are your perceptions about the use of these resources which were allocated to RapidSMS?

III. RapidSMS relevance

10. How was the adoption and continued use of RapidSMS related to national or international development targets, including MDGs, Vision 2020, EDPRS 2, and SDGs?

11. To what extent is RapidSMS data used in planning and decision-making?

12. Can you think of areas where RapidSMS is not currently having an impact where it could be?

Probe : For each area identified, how would RapidSMS have to change for such an impact to be made?

IV. Impact of RapidSMS

13. What impact do you believe the RapidSMS system has had on health in Rwanda?

Probe: What impact do you believe it has had on maternal health?
Probe: What impact do you believe it has had on Newborn health?
Probe: What impact do you believe it has had on Child health?

14. How has RapidSMS contributed to the reduction of maternal, newborn, and child mortality?

V. RapidSMS effectiveness and efficiency

15. What changes in maternal, newborn and child health services were prompted by RapidSMS?

Probe 1: Any change in the maternal, newborn and child continuum of care?
Probe 2: Any change in access to and utilization of these services?
Probe 3: Any change in providers' knowledge, skills and practices?

VI. RapidSMS coordination and sustainability

16. How is the RapidSMS system coordinated and managed?

17. What are your views regarding how well this coordination and management structure is functioning?

Probe: What elements of the coordination/management should be kept?

Probe: What elements could be improved?

18. How is the government including/integrating RapidSMS in the Government policies, legislation, strategies, information systems and budgets?

19. To what extent has RapidSMS helped in managing maternal, newborn and child health more efficiently?

20. What challenges do you think RapidSMS will face in terms of sustainability?

Probe 1: How these challenges can be mitigated?

21. What do you think the role of your institution could be in ensuring the sustainability of RapidSMS?

Closing

Thank you very much for taking your precious time to discuss the RapidSMSprogram with us.

Is there any information you would like to share with us that did not get covered in this interview?

We may be in touch again in case there is need for clarification or to ask about something that was not discussed but emerged as important during transcription and analysis of the data. Would you be available for such follow-up?

Thank you so much for agreeing to follow-up.

Interview Guide

Academia and National Professional Society personnel

Project Title: Evaluating the Impact of RapidSMS

Principal Investigator: Michael Law, PhD

University of Rwanda-College of Medicine and Health Sciences/ School of Public Health, Kigali

Respondent Information

1. Name:
2. Organization:
3. Title (describe his/her role):
   ……
   ………………………………………………………
   …………………………………………………………………………………
4. Gender:
5. Level of education:
6. Present position:
7. Number of years and Experience at the current position:
8. Previous organization where s/he worked, position, and number of years and roles:

   ……

General topics to be discussed during the interview

I. Introduction questions

1. What are your responsibilities related to maternal and child health?

2. To what extent are you familiar with the RapidSMS system?

3. How were you involved in the development of the RapidSMS system?

II. RapidSMS design and implementation

4. What problems do you think the RapidSMS system in Rwanda was intended to address?

5. What is your institution's role in the RapidSMS system?

Probe: Do you participate in the analysis of RapidSMS data?

6. What are your perceptions about the amount of resources that were allocated to the RapidSMS system?

7. What are your perceptions about the use of the resources which were allocated to the RapidSMS?

III. Impact of RapidSMS

8. What impact do you believe the RapidSMS system has had on health in Rwanda?

Probe: What impact do you believe it has had on maternal health?
Probe: Newborn health?
Probe: Child health?

9. What specific changes to academic curricula or continuing professional development do you think have occurred due to the RapidSMS system?

10. Can you think of areas where RapidSMS is not currently having an impact where it could be?

Probe 1: For each area identified, how would RapidSMS have to change for such an impact to be made?

IV. RapidSMS effectiveness and efficiency

11. What changes in maternal, newborn and child health services were promptedby RapidSMS?

Probe 1: Any change in the maternal, newborn and child continuum of care?
Probe 2: Any change in access to and utilization of these services?
Probe 3: Any change inproviders' knowledge, skills and practices?

12. To what extent has RapidSMS helped in managing maternal, newborn and child health more efficiently?

V. RapidSMS coordination and sustainability

13. What are the challenges that you foresee RapidSMS facing in terms of sustainability?

14. How can these challenges be mitigated?

15. What do you think the role of your institution could be in ensuring the sustainability of RapidSMS?

Closing

Thank you very much for taking your precious time to discuss this program with us.

Is there any information you would like to share with me that did not get covered in this interview?

I may be in touch again in case there is need for clarification or to ask about something that was not discussed but emerged as important during transcription and analysis of the data. Would you be available for such follow-up?

Thank you so much for agreeing to follow-up.

Interview Guide

District and Health Facility Authorities

Research Project: Evaluating the impact of RapidSMS

Principal Investigator: Michael Law, PhD

University of Rwanda-College of Medicine and Health Sciences/ School of Public Health, Kigali

Respondent Information

1. Name:
2. Organization:
3. Title (describe his/her role): ……
4. Gender:
5. Level of education:
6. Present position:
7. Number of years and Experience at the current position:
8. Previous organization where s/he worked, position, and number of years and roles: ……

General topics to be discussed during the interview

I. Introduction questions

1. What are your responsibilities related to maternal and child health?

2. How were you involved in the development and implementation of the RapidSMS system?

II. RapidSMS design and implementation

3. What do you think the major objectives of RapidSMS were when it started?

4. How were decisions made about where and when to implement RapidSMS?

Probe: How did equity considerations play a role in these decisions?

5. What, if any, challenges were faced in implementing the RapidSMS system? How were they overcome?

6. How did you consult and collaborate with local leaders in implementing RapidSMS?

7. What are your perceptions about the amount of human and financial resources that were allocated

to the RapidSMS?

8. What are your perceptions about the use of these resources which were allocated to the RapidSMS?

9. What do you think could be improved in the way that RapidSMS is implemented and used?

Probe: in terms of capacity building?

10. How can these improvements be performed?

11. Who should be involved in implementing these improvements?

III. RapidSMS relevance

12. How well was RapidSMS accepted by your community, including both community health workers and the general population?

Probe: Did it fit your community priorities?

13. Which group(s) of beneficiaries has/have been reached by RapidSMS? Are there groups that are being missed?

14. To what extent was and is RapidSMS data used in decision-making?

IV. Impact of RapidSMS

15. How do you think RapidSMS impacted maternal, child, and newborn health in your area?

Probe: Can you share a specific example with me?

16. What are any other changes you think resulted from the implementation of the RapidSMS system?

17. What challenges have you face in using the RapidSMS system?

18. Can you think of areas where RapidSMS is not currently having an impact where it could be?

Probe 1: For each area identified, how would RapidSMS have to change for such an impact to be made?

V. RapidSMS effectiveness and efficiency

19. What changes in planning, providing, and monitoring maternal, newborn, and child health services were shaped by RapidSMS?

Probe 1: Any change in the maternal, newborn and child continuum of care?
Probe 2: Any change in access to and utilization of these services?
Probe 3: Any change inproviders' knowledge, skills and practices on essential maternal and newborn care?

Probe:what implications do you think this has for the sustainability of the program?

20. To what extent has RapidSMS helped in managing maternal, newborn and child health more efficiently?

VI. RapidSMS coordination and sustainability

21. What are your views regarding the coordination and management of the RapidSMS system?

Probe: What elements of the coordination/management could be kept up?

Probe: What elements could be improved?

22. How do Districts and Health Facilities demonstrate their ownership of the RapidSMS program?

Closing

Thank you very much for taking your precious time to discuss RapidSMS with us

Is there any information you would like to share with us that did not get covered in this interview?

We may be in touch again in case there is need for clarification, amplification, or to ask about something that was not discussed but emerged as important during transcription and analysis of the data. Would you be available for such follow-up?

Thank you so much for agreeing to follow-up.

Interview Guide

Maternity and Community Health Staff

Research Project: Evaluating the Impact of RapidSMS

Principal Investigator: Michael Law, PhD

University of Rwanda-College of Medicine and Health Sciences/ School of Public Health, Kigali

<u>Respondent Information</u>

1. Name:
2. Organization:
3. Title (describe his/her role): ………
4. Gender:
5. Level of education:
6. Present position:
7. Number of years and Experience at the current position:
8. Previous organization where s/he worked, position, and number of years and roles:

   ………

I. <u>General topics to be discussed during the interview</u>

II. Introduction questions

1. What are your responsibilities related to maternal and child Health?

III. RapidSMS design and implementation

2. What role does the RapidSMS system play in your day-to-day work?

3. What, if any, challenges were faced in implementing the RapidSMS system in your facility? How were they overcome?

IV. RapidSMS relevance

4. To what extent is RapidSMS data used in decision-making within your facility?

5. Which group(s) of beneficiaries have been reached by RapidSMS? Are there groups that are being missed?

6. How well was RapidSMS accepted by your community, including both your community health workers and the general population?

Probe: Did it fit your community priorities?

V. Impact of RapidSMS

7. How do you think RapidSMS impacted maternal, child, and newborn health in your area?

Probe: Can you share a specific example with me?

8. How do you think RapidSMS has changed the way that mothers and their children interact with the health system?

9. What are any other changes you think have occurred due to the implementation of the RapidSMS system?

10. Can you think of areas where RapidSMS is not currently having an impact where it could be?

Probe 1: For each area identified, how would RapidSMS have to change for such an impact to be made?

VI. RapidSMS effectiveness and efficiency

11. What changes in planning, providing, and monitoring maternal, newborn and child health services were shaped by RapidSMS?

Probe 1: Any change in the maternal, newborn and child continuum of care?
Probe 2: Any change in access to and utilization of these services?
Probe 3: Any change CHWs' knowledge, skills and practices on essential maternal and newborn care at their level?

12. What are your perceptions about the amount of resources which were allocated to RapidSMS?

13. What are your perceptions about the use of resources which were allocated to RapidSMS?

VII. RapidSMS coordination and sustainability

14. What are your views regarding the coordination and management of the RapidSMS system?

Probe: What elements of the coordination/management could be kept up?

Probe: What elements could be improved?

15. How committed and motivated do you think CHWs are to continue the use of RapidSMS in the future? Why?

Probe: What could be changed to make them more motivated or committed?

16. What do you think could be improved in the way that RapidSMS is implemented?

17. How can these improvements be performed?

18. Who should be involved in implementing these improvements?

Closing

Thank you very much for taking your precious time to discuss RapidSMS with us

Is there any information you would like to share with me that did not get covered in this interview?

I may be in touch again in case there is need for clarification, amplification, or to ask about something that was not discussed but emerged as important during transcription and analysis of the data. Would you be available for such follow-up?

Thank you so much for agreeing to follow-up.

Interview Guide

Cell Coordinator

Project Title: Evaluating the Impact of RapidSMS Rwanda

Principal Investigator: Michael Law, PhD

University of Rwanda-College of Medicine and Health Sciences/ School of Public Health, Kigali

Respondent Information

1. Name:
2. Organization:
3. Title (describe his/her role): ………………………………………………………………………………………………………
4. Gender:
5. Level of education:
6. Present position:
7. Number of years and Experience at the current position:

I. Introduction Questions

1. As a CHW, what is your role in maternal and child health?

2. How were you and the people you supervise trained about the use of the RapidSMS system?

3. What are your perceptions about the quality of this training? In what ways was this training adequate, and in what ways was it lacking?

4. Do you feel that the refresher trainings are enough to keep you up to date on the RapidSMS system? What about them could be improved?

II. RapidSMS design and implementation

5. What role does RapidSMS play in your daily work?

6. What challenges have you faced using RapidSMS? How have you overcome these challenges?

III. RapidSMS relevance

7. What role has RapidSMS had in achieving your district-level targets?

8. To what extent has RapidSMS data been used in decision-making at the District/sector/cell/village

level?

IV. Impact of RapidSMS

9. Which group(s) of beneficiaries has/have been reached by RapidSMS? Are there groups that are being missed?

10. Do you think that RapidSMS is as useful after birth as before? Why?

11. Which beneficiaries do you think are reached by RapidSMS?

12. What impact do you believe the RapidSMS system has had on health in your area?

Probe: What impact do you believe it has had on maternal health?

Probe: What impact do you believe it has had on Newborn health?

Probe: What impact do you believe it has had on Child health?

13. Can you think of areas where RapidSMS is not currently having an impact where it could be?

Probe 1: For each area identified, how would RapidSMS have to change for such an impact to be made?

V. RapidSMS effectiveness and efficiency

14. What are your perceptions about the amount of resources and materials which are allocated to the RapidSMS system?

15. What are your perceptions about the use of the resources and materials which are allocated to the RapidSMS system?

16. What changes in planning, providing, and monitoring maternal, newborn, and child health services were shaped by RapidSMS?

Probe 1: Any change in the maternal, newborn and child continuum of care?
Probe 2: Any change in access to and utilization of these services?
Probe 3: Any change in providers' knowledge, skills and practices on essential maternal and newborn care?

VI. RapidSMS coordination and sustainability

17. How has the commitment and motivation of CHWs been fostered to continue theiruse of RapidSMS in the future?

18. What support do you need in order to continue or improve your use of the RapidSMS system?

19. What are the challenges that you foresee RapidSMS facing in terms of sustainability?

20. How can these challenges be mitigated?

Closing

Thank you very much for taking your precious time to discuss the RapidSMS system with us.

Is there any information you would like to share with me that did not get covered in this interview?

We may be in touch again in case there is need for clarification or to ask about something that was not discussed but emerged as important during transcription and analysis of the data. Would you be available for such follow-up?

Once again thank you.

Ikiganiro kigenewe umuhuzabiorwa w'abajyanama b'ubuzima mu Kagari

Izina ry'ubushakashatsi: Gusuzuma impinduka zatewe na RapidSMS mu Rwanda

Umuyobozi mukuru w'ubushakashatsi: Michael Law, PhD

Kaminuza y'uRwanda- Koleji y'ubuvuzi n'ubumenyi mu by'ubuzima- Ishuri ry'ubuzima rusange Kigali

<u>Amakuru yerekeye ubazwa</u>

1. Amazina:
2. Icyo akora (vuga akazi ke): ………
3. Igitsina:
4. Amashuri yize:
5. Imyaka amaze ku nshingano afite ubu:

I. Ibibazo rusange bibanza

1. Nk'umujyanama w'ubuzima, ni uruhe ruhare ugira mu kubungabunga ubuzima bw'umwana n'umubyeyi?

2. Wowe ubwawe n'abantu ureberera, ni mu buhe buryo mwahuguriwe gukorasha RapidSMS?

3. Ayo mahugurwa mwahawe uyabona mute? Ni ku zihe ngingo mubona aya mahugurwa yari afite ingufu kandi ni iki mubona yari abuze?

4. Mbese ubona amahugurwa yo kwiyibutsa muhabwa ahagije kugira ngo mujyane n'igihe kandi mumenye ibigezweho mu gukoresha RapidSMS? Mubona ari iki cyanozwa biruseho ku bijyanye n'aya mahugurwa yo kwiyibutsa?

II. Iringanizwa n'ishyirwa mu bikorwa rya RapidSMS

5. Ni uruhe ruhare gahunda ya RapidSMS igira mu mirimo yanyu ua buri munsi?

6. Ni izihe nzitizi mwaba mwarahuye na zo mu gukoresha RapidSMS? Ni ubuhe buryo mwifashishije kugira ngo murenge izo nzitizi?

III. Ukuba ingenzi kwa RapidSMS

7. Ni uruhe ruhare RapidSMS yagize mu kwesa imihigo y'akarere kanyu?

8. Ni ku ruhe rwego ibiva muri gahunda ya RapidSMS byifashishwa mu gufata imyanzuro ku rwego rw"Akarere/Umurenge/Akagari/Umudugudu?

IV. Impinduka zazanywe na RapidSMS

9. Ni ayahe matsinda yagiriwe umumaro na RapidSMS? Hari amatsinda itagiriye umumaro kandi byari byitezwe ko iyi gahunda iyageraho?

10. Mutekereza ko RapidSMS igira akamaro nyuma yo kubyara nk'uko ikagira na mbere y'aho? Kubera iki?

11. Ni izihe mpinduka ku buzima bw'abantu b'aho muyobora mutekereza ko zazanywe na gahunda ya RapidSMS?

Baza n'iki: Ni izihe mpinduka mutekereza ko yazanye ku buzima bw'ababyeyi?

Baza n'iki: Ni izihe mpinduka mutekereza ko yazanye ku buzima bw'abana bakivuka?

Baza n'iki: Ni izihe mpinduka mutekereza ko yazaniye ku buzima bw'abana barengeje iminsi 28?

12. Hari ibyo mubona RapidSMS itagize icyo ibihinduraho kandi yari yitezweho kubizanamo impinduka?

Baza n'iki: Ni mu buhe buryo RapidSMS yahindurwamo kugira ngo izo mpinduka zitezwe zigerweho?

V. RapidSMS effectiveness and efficiency

13. Ni gute mubona amafaranga, imbaraga n'ibikoresho bigenerwa gahunda RapidSMS?

14. Ni izihe mpinduka zaba zarazanywe na Rapid SMS mu bijyanye n'igenabikorwa, gutanga no gukurikirana imitangire ya serivisi z'ubuzima/ubuvuzi zigenerwa ababyeyi n'abana?

Baza n'iki: Hari impinduka zagaragaye mu gukomerezaho kwa serivisi z'ubuzima/ubuvuzi zigenerwa ababyeyi n'abana?
Baza n'iki: Hari impinduka zagaragaye mu buryo izi serivisi zitabirwa?
Baza n'iki: Hari impinduka zagaragaye mu bumenyi, bushobozi n'imikorere by'abavuzi mu buvuzi bakorera ababyeyi n'abana bakivuka?

VI. Imicungire ya RapidSMS n'Ahazaza hayo

15. Ni gute ubwuzu n'ubushake byubatswe mu bajyanama b'ubuzima kugira ngo bashishikarire kuzakomeza gukoresa RapidSMS mu gihe kizaza?

16. Ni iyhe nkunga mwumva mukeneye kugira ngo muzakomeze gukoresha no kunoza gahunda ya RapidSMS mu gihe kizaza?

17. Ni izihe mnzitizi mubona RapidSMS yazahura na zo bigatuma idakomeza gukoreshwa mu gihe kizaza?

18. Mubona hakorwa iki kugira ngo izi nzitizi zikurweho?

Gusoza

Turabashimira cyane kubw'igihe cyanyu mwaduhaye mukadusangiza uko mubona gahunda ya RapidSMS.

Hari amakuru y'ingenzi ajyanye na RapidSMS tutaganiriyeho mwaba muifuza kudusangiza mbere y'uko dusoza?

Dushobora gukenera kuzongera kubavugisha turamutse hari icyo tudasobanukiwe mu byo mwatubwiye, cyangwa tukababaza ibindi bibazo tutaganiriyeho igihe tuzasanga ari ingenzi turi kwandikura ibyo twaganiriye. Mbese mwabitwemerera?

Twongeye kubashimira ubitange bwanyu.

ASM-CHWs FGD Agenda

Project Title: Evaluating the Impact of RapidSMS Rwanda

Principal Investigator: Michael Law, PhD

University of Rwanda-College of Medicine and Health Sciences/ School of Public Health, Kigali

(For FGD moderator and note taker: Remember to complete the attached participant log)

I. Introduction Questions

1. What role does the RapidSMS system play in your day-to-day work?

2. How were you trained about the use of the RapidSMS system?

3. What are your perceptions about the quality of this training? In what ways was this training adequate, and in what ways was it lacking?

4. Do you feel that the refresher trainings are enough to keep you up to date on the RapidSMS system? What about them could be improved?

II. RapidSMS design and implementation

5. What challenges, if any, have you faced in using the RapidSMS system?

6. What methods, if any, have you used to overcome these challenges?

7. What groups in your village are vulnerable for poor pregnancy and early childhood outcomes?

Probe: What has been done to assist these people? Could something be improved?

Probe: What challenges do they face in benefiting from RapidSMS?

8. Are there any other groups in your village who are not benefitting from RapidSMS services?

Probe: Ask about youth, single mothers, religious groups, MNH services, etc.

III. RapidSMS relevance

9. What role has RapidSMS played in achieving your district-level targets?

IV. Impact of RapidSMS

10. How well do the reminders you receive from RapidSMS work in changing health care use by mothers in your village?

11. How do you think RapidSMS has impacted maternal, child, and newborn health in your village?

Probe: Can you share a specific example with me?

12. Do you think that RapidSMS is as useful after birth as before? Why?

13. How has RapidSMS changed how you provide care? Do you think it has improved your knowledge, skills, and practices as a CHW?

14. What economic impact has RapidSMS had on you and your family?

Probe: Does this financial impact provide motivation to continue using RapidSMS?

15. Can you think of areas where RapidSMS is not currently having an impact where it could be?

Probe 1: For each area identified, how would RapidSMS have to change for such an impact to be made?

V. RapidSMS effectiveness and efficiency

16. What role has RapidSMS played, if any, in changing use of the health care system by mothers in your village?

17. What are your perceptions about the amount of resources and materialswhich are allocated to the RapidSMS system?

VI. RapidSMS coordination and sustainability

18. To what extent is the time involved in using the RapidSMS worth the effort?

19. What support do you need in order to continue or improve your use of the RapidSMS system?

Closing

Thank you very much for taking your precious time to discuss the RapidSMS system with us.

Is there any information you would like to share with me that did not get covered in this interview?

Once again thank you.

IKIGANIRO CY'ABAJYANAMA B'UBUZIMA (ASM)

Izina ry'ubushakashatsi: Gusuzuma impinduka zatewe na RapidSMS mu Rwanda

Umuyobozi mukuru w'ubushakashatsi: Michael Law, PhD

Kaminuza y'uRwanda- Koleji y'ubuvuzi n'ubumenyi mu by'ubuzima- Ishuri ry'ubuzima rusange, Kigali

(UMUYOBOZI W'IKIGANIRO N'UMWANDITSIWE:MWIBUKE KUZUZA IFISHI Y'IMYIRONDORO Y'ABAGIZE URUHARE MURI IKI KIGANIRO)

I. Ibibazo rusange bibanza

1. Ni uruhe ruhare gahunda ya RapidSMS igira mu mirimo yanyu ua buri munsi?

2. Ni mu buhe buryo mwahuguriwe gukorasha RapidSMS?

3. Ayo mahugurwa mwahawe muyabona mute? Ni ku zihe ngingo mubona aya mahugurwa yari afite ingufu kandi ni iki mubona yari abuze?

4. Mbese mubona amahugurwa yo kwiyibutsa muhabwa ahagije kugira ngo mujyane n'igihe kandi mumenye ibigezweho mu gukoresha RapidSMS? Mubona ari iki cyanozwa biruseho ku bijyanye n'aya mahugurwa yo kwiyibutsa?

II. Iringanizwa n'ishyirwa mu bikorwa rya RapidSMS

5. Ni izihe nzitizi –niba zihari- mwaba mwarahuye na zo mu gukoresha RapidSMS?

6. Ni ubuhe buryo -niba buhari- mwifashishije kugira ngo murenge izo nzitizi?

7. Mu mudugudu wanyu ni ayahe matsinda y'abantu yaba afite ingorane zo guhura n'ibibazo mu gihe batwite cyangwa abana babo bakaba bagira ibibazo bakiri bato?

-Baza n'iki: Ni iki cyakozwe kugira ngo aba bantu bafashwe? Mubona hari igikwiriye konozwa kurushaho kubwabo?

-Baza n'iki: Ni izihe mbogamizi bahura na zo zituma batabonera ubufasha muri gahunda ya RapidSMS?

8. Hari ayandi matsinda yaba adakoresha serivisi za RapidSMS zitangwa n'abajyanama b'ubuzima cyangwa n'ikigo nderabuzima?

- **Baza n'iki:** Urubyiruko, Abakobwa babyaye, religioamatsinda ashingiye ku madini?

III. Ukuba ingenzi kwa RapidSMS

9. Ni uruhe ruhare RapidSMS yagize mu kwesa imihigo y'akarere kanyu?

IV. Impinduka zazanywe na RapidSMS

10. Ni mu buhe buryo ubutumwa bwo kwibutsa bwa RapidSMS mwakira bugira uruhare mu guhindura ukuntu ababyeyi bo mu mudugudu wanyu bitabira ubuvuzi bagenerwa?

11. Mubona mute uburyo RapidSMS yazanye impinduka ku buzima bw'ababyeyi n'abana bo mu mudugudu wanyu?

Probe: Mwaduha ingero zifatika?

12. Mutekereza ko RapidSMS igira akamaro nyuma yo kubyara nk'uko ikagira na mbere y'aho? Kubera iki?

13. Nk'abajyanama, ni mu buhe buryo RapidSMS yahinduye uko mutanga servisi z'ubuzima? Mutekereza ko iyi gahunda yabongereye ubumenyi, ubushobozi igahindura n'imikorere yanyu?

14. Ni izihe mpinduka mu by'ubukungu RapidSMS yakuzaniye wowe ubwawe no ku muryango wawe?

Baza n'iki: Izo mpinduka se zituma mwumva mwakomeza gukoresha RapidSMS?

15. Hari ibyo mubona RapidSMS itagize icyo ibihinduraho kandi yari yitezweho kubizanamo impinduka?

Baza n'iki:: Ni mu buhe buryo RapidSMS yahindurwamo kugira ngo izo mpinduka zitezwe zigerweho?

V. Imbaraga RapidSMS isaba n'umusaruro itanga

16. Ni uruhe ruhare -niba ruhari-, RapidSMS yagize mu guhindura uburyo ababyeyi bo mu mudugudu wanyu bakoreshamo serivisi z'ubuzima?

17. Ni gute mubona amafaranga, imbaraga n'ibikoresho bigenerwa gahunda RapidSMS?

VI. Imicungire ya RapidSMS n'Ahazaza hayo

18. Mubona igihe mufata mukoreshsa RapidSMS gikwiranye n'akamaro kayo?

19. Ni iyhe nkunga mwumva mukeneye kugira ngo muzakomeze gukoresha no kunoza gahunda ya RapidSMS mu gihe kizaza?

Gusoza

Turabashimira cyane kubw'igihe cyanyu mwaduhaye mukadusangiza uko mubona gahunda ya RapidSMS.

Hari amakuru y'ingenzi ajyanye na RapidSMS tutaganiriyeho mwaba muifuza kudusangiza mbere y'uko dusoza?

Twongeye kubashimira ubitange bwanyu.

Mothers FGD Agenda

Project Title: Evaluating the Impact of RapidSMS Rwanda

Principal Investigator: Michael Law, PhD

University of Rwanda-College of Medicine and Health Sciences/ School of Public Health, Kigali

(For FGD moderator and note taker: Remember to fill the participant information sheet)

I. Introduction Questions

1. In your village, once a woman is pregnant, who is typically the first person she consults for getting care?

2. What isthe role of the ASM-CHW in your village with respect to pregnancy, child birth, and newborns?

3. What challenges do you face in accessing the health care system?

4. What do you know about the RapidSMS system?

Probe: Describe the RapidSMS system if they do not know of it.

II. RapidSMS design and implementation

5. How were you informed about the RapidSMS system?

6. What is the importance of RapidSMS in your community?

III. Impact of RapidSMS

7. How did RapidSMS change the way that you interacted with the health system?

Probe: For those who have given birth previously, how did RapidSMS change your experience?

8. What impact do you believe the RapidSMS system has had on your pregnancy and the care you provided for your child?

IV. RapidSMS effectiveness and efficiency

V. RapidSMS sustainability

9. What do you think could be changedabout the RapidSMS system to bring more benefit to yourself or your community?

10. How can these improvements be performed?

11. Who should be involved in implementing these improvements?

Closing

Thank you very much for taking your precious time to share your personal experience with the RapidSMS system with us.

Is there any information you would like to share with me that did not get covered in this interview?

Once again thank you so much.

IKIGANIRO CY'ABAGENERWABIKORWA (ABAGORE)

Izina ry'ubushakashatsi: Gusuzuma impinduka zatewe na RapidSMS mu Rwanda

Umuyobozi mukuru w'ubushakashatsi: Michael Law, PhD

Kaminuza y'uRwanda- Koleji y'ubuvuzi n'ubumenyi mu by'ubuzima- Ishuri ry'ubuzima rusange, Kigali

(UMUYOBOZI W'IKIGANIRO N'UMWANDITSIWE: MWIBUKE KUZUZA IFISHI Y'IMYIRONDORO Y'ABAGIZE URUHARE MURI IKI KIGANIRO)

I. <u>Ibibazo rusange bibanza</u>

1. Mu mudugudu wanyu, iyo umugore atwite ni nde muntu wa mbere asanga kugira ngo amuhe ubufasha mu rwego rw'ubuvuzi?

2. Umujyanama w'ubuzima-ASM, afite izihe nshingano mu mudugudu wanyu ku bagore batwite, ukubyara kwabo no ku mpinja zivuka?

3. Ni izihe mbogamizi/inzitizi muhura na zo zishobora gutuma mutagera kuri serivisi z'ubuvuzi uko bikwiriye?

4. Ni iki mwaba muzi kuri gahunda ya RapidSMS?

(BASOBANURIRE GAHUNDA YA RapidSMS NBA BATAYIZI).

II. <u>Iringanizwa n'ishyirwa mu bikorwa rya RapidSMS</u>

5. Ni gute mwamenyeshejwe gahunda ya RapidSMS?

6. Aho mutuye, ni iki iyi gahunda ya RapidSMS ibamariye?

III. <u>Impinduka zazanywe na RapidSMS</u>

7. Ni mu buhe buryo RapidSMS yaba yarahinduye uburyo mukorana n'inzego z'ubuvuzi/zishinzwe ubuzima?

Baza n'iki: Ku bagore babyaye mbere y'uko gahunda ya RapidSMS itangira, ni gute iyi gahunda yaba yarahinduye uko byajyaga bibagendekera mbere?

8. Ni izihe mpinduka ku migendekere yo gutwita kwanyu no ku buryo abana banyu bitabwaho mutekereza ko zazanywe na gahunda ya RapidSMS?

IV. Ahazaza ha gahunda ya RapidSMS

9. Ni ibiki mutekereza ko bikwiriye kunozwa biruseho muri gahunda ya RapidSMS kugira ngo mwebwe ubwanyu n'abaturanyi banyu muyibonereho ubufasha busumbyeho?

10. Ni mu buhe buryo mubona izo mpinduka zashyirwa mu bikorwa?

11. Ni bande mutekereza ko bakwiriye kugira uruhare mu kunoza iyi gahunda bashyira mu bikorwa izo mpinduka?

Gusoza

Turabashimira cyane kubw'igihe cyanyu mwaduhaye mukadusangiza uko mubona gahunda ya RapidSMS.

Hari amakuru y'ingenzi ajyanye na RapidSMS tutaganiriyeho mwaba muifuza kudusangiza mbere y'uko dusoza?

Twongeye kubashimira ubitange bwanyu.

Fathers FGD Agenda

Project Title: Evaluating the Impact of RapidSMS Rwanda

Principal Investigator: Michael Law, PhD

University of Rwanda-College of Medicine and Health Sciences/ School of Public Health, Kigali

(For FGD moderator and note taker: Remember to fill the participant information sheet)

I. Introduction Questions

1. In your village, once a woman is pregnant, who is typically the first person she consults for getting care?
2. In your village, once a woman is pregnant who take a decision about who to consult?
3. What is the role of the ASM-CHW in your village with respect to pregnancy, child birth, and newborns?
4. As a father, what is your role in maternal and child health?
5. What challenges do you face in accessing the health care system?
6. What do you know about the RapidSMS system?

   Probe: Describe the RapidSMS system if they do not know of it.

II. RapidSMS design and implementation

7. How were you informed about the RapidSMS system?
8. What is the importance of RapidSMS in your community?

III. Impact of RapidSMS

9. How did RapidSMS change the way that you and your wife interacted with the health system?

   Probe : For those whose wife have given birth previously, how did RapidSMS change your experience?

10. What impact do you believe the RapidSMS system has had on the pregnancy of your wife and the care you or your wife provided for your child?

IV. RapidSMS sustainability

11. What do you think could be changed about the RapidSMS system to bring more benefit to yourself, your wife, children or your community?

12. How can these improvements be performed?

13. Who should be involved in implementing these improvements?

Closing

Thank you very much for taking your precious time to share your personal experience with the RapidSMS system with us.

Is there any information you would like to share with me that did not get covered in this interview?

Once again thank you so much.

IKIGANIRO CY'ABAGENERWABIKORWA (ABAGABO)

Izina ry'ubushakashatsi: Gusuzuma impinduka zatewe na RapidSMS mu Rwanda

Umuyobozi mukuru w'ubushakashatsi: Michael Law, PhD

Kaminuza y'uRwanda- Koleji y'ubuvuzi n'ubumenyi mu by'ubuzima- Ishuri ry'ubuzima rusange, Kigali

(UMUYOBOZI W'IKIGANIRO N'UMWANDITSIWE: MWIBUKE KUZUZA IFISHI Y'IMYIRONDORO Y'ABAGIZE URUHARE MURI IKI KIGANIRO)

I. Ibibazo rusange bibanza

1. Mu mudugudu wanyu, iyo umugore atwite ni nde muntu wa mbere asanga kugira ngo amuhe ubufasha mu rwego rw'ubuvuzi?

2. Mu mudugudu wanyu, iyo umugore atwite ni nde ufata umwanzuro w'uwo akwiriye gusanga ngo amugire inama?

3. Umujyanama w'ubuzima-ASM, afite izihe nshingano mu mudugudu wanyu ku bagogre batwite, ukubyara kwabo no ku mpinja zivuka?

4. Nk'umubyeyi, ni uruhe ruhare mugira mu buzima bw'abana n'ababyeyi?

5. Ni izihe mbogamizi/inzitizi muhura na zo zishobora gutuma mutagera kuri serivisi z'ubuvuzi uko bikwiriye?

6. Ni iki mwaba muzi kuri gahunda ya RapidSMS?

(BASOBANURIRE GAHUNDA YA RapidSMS NBA BATAYIZI).

II. Iringanizwa n'ishyirwa mu bikorwa rya RapidSMS

7. Ni gute mwamenyeshejwe gahunda ya RapidSMS?

8. Aho mutuye, ni iki iyi gahunda ya RapidSMS ibamariye?

I. Impinduka zazanywe na RapidSMS

9. Ni mu buhe buryo RapidSMS yaba yarahinduye uburyo mwebwe n'abo mwashakanye mukorana n'inzego z'ubuvuzi/zishinzwe ubuzima?

Baza n'iki: Ku bagabo bafite abagore babyaye mbere y'uko gahunda ya RapidSMS itangira, ni gute iyi gahunda yaba yarahinduye uko byajyaga bibagendekera mbere?

10. Ni izihe mpinduka ku migendekere yo gutwita k'umugore wanyu no ku buryo abana banyu bitabwaho mutekereza ko zazanywe na gahunda ya RapidSMS?

III. Ahazaza ha gahunda ya RapidSMS

11. Ni ibiki mutekereza ko bikwiriye kunozwa biruseho muri gahunda ya RapidSMS kugira ngo mwebwe ubwanyu, abo mwashakanye, abana banyu n'abaturanyi banyu muyibonereho ubufasha busumbeho?

12. Ni mu buhe buryo mubona izo mpinduka zashyirwa mu bikorwa?

13. Ni bande mutekereza ko bakwiriye kugira uruhare mu kunoza iyi gahunda bashhyira mu bikorwa izo mpinduka?

<u>Gusoza</u>

Turabashimira cyane kubw'igihe cyanyu mwaduhaye mukadusangiza uko mubona gahunda ya RapidSMS.

Hari amakuru y'ingenzi ajyanye na RapidSMS tutaganiriyeho mwaba muifuza kudusangiza mbere y'uko dusoza?

Twongeye kubashimira ubitange bwanyu.
